# Getting Back on the Path to Eliminating HIV in Children

**DOI:** 10.1002/jia2.70102

**Published:** 2026-06-19

**Authors:** George K. Siberry

**Affiliations:** ^1^ Department of Pediatrics Columbia University Irving Medical Center New York New York USA

**Keywords:** elimination, paediatric HIV, vertical transmission

## Abstract

**Introduction:**

The global community proved remarkably resilient in protecting the progress in and commitment to eliminating HIV in children in the face of the COVID‐19 pandemic. However, sudden disruptions in 2025 in donor support for HIV assistance have posed new and existential threats to the hard‐fought gains in global paediatric HIV control.

**Discussion:**

Since the early 2000s, the reductions in paediatric HIV acquisitions, in deaths in children with HIV and in the number of children orphaned by HIV/AIDS have been breathtaking, but in recent years, progress has stalled, and gaps remain. The disruptions in political and financial support from the U.S. government obligate us to reframe our plans for ending HIV as a threat to children and also enable us to implement overdue changes in strategy to overcome barriers to success. Pillars for success include: robust integration of HIV into strengthened general healthcare systems; reassertion of national government leadership; prioritizing reduced donor funding for research and innovation; stronger partnership between private sectors and public health programmes; amplified community voices; and bold determination and creative solutions from global paediatric HIV stakeholders.

**Conclusions:**

The time is right for the global paediatric HIV community to consider the key factors that have enabled the tremendous progress towards ending the threat of HIV in children, the critical barriers to overcoming remaining gaps and a framework no longer so dependent on donor support for getting back on the path to eliminating HIV in children. The diverse and talented global HIV stakeholder coalition will find the opportunities for transformation and innovation in paediatric HIV care and boldly chart a lasting, evidence‐based, person‐centred, community‐informed, national health system‐aligned path to ending HIV as a threat to children.

## 1 Introduction

The progress in the past 20–30 years towards ending the threat of HIV/AIDS to children worldwide has been astounding: annual new paediatric acquisitions declined from over 500,000 to less than 100,000; AIDS‐related deaths in children dropped to under 75,000 after peaking at nearly 400,000; and the number of children orphaned by HIV/AIDS is now decreasing by 800,000 per year instead of increasing by up to 1.3 million per year. COVID‐19 threatened to upend these remarkable gains, but the global HIV community and especially country‐led HIV programming proved remarkably resilient and resourceful in mitigating the disruptions to HIV services for pregnant and breastfeeding women and their children [1]. The U.S. government's abrupt withdrawal from the HIV and global health response, however, poses an existential threat to ending HIV in children. But our commitment to HIV‐free generations of children only strengthens our resolve to chart a successful new path to eliminating vertical HIV transmission and paediatric AIDS in a transformed context that depends less on donor assistance.

## 2 Discussion

### 2.1 Global Progress in Protecting Children From HIV

Just 30 years ago, we were helpless to prevent HIV acquisitions in 25% (up to 50% with breastfeeding) of infants born to women living with HIV. Without effective prevention interventions and scalable treatment, massive numbers of children suffered from acquiring HIV (and death) and staggering numbers of children lost one or both parents to HIV/AIDS, especially in sub‐Saharan Africa. HIV‐driven economic crises and the decimation of working‐age adult populations threatened entire populations in many African countries [2].

The robust, coordinated global response to the devastating HIV pandemic, propelled by the launch of PEPFAR and Global Fund in the early 2000s, produced breathtaking and inspiring progress: Annual paediatric HIV acquisitions that peaked at over 550,000 at the turn of the century were cut in half by 2011 and in half again by 2022. The global vertical transmission rate was slashed from 23% in 2010 to 10% in 2024. The number of children with HIV declined from 2.9 million in 2005 to 1.4 million in 2024. AIDS‐related deaths in children dropped from 380,000 in 2002 to 75,000 in 2024 [3].

Antiretroviral therapy (ART) for pregnant women with HIV—directly lifesaving for women and the most important intervention to prevent vertical transmission—reached 50% of women globally by 2010, exceeded 75% by 2014 and reached 84% in 2019 [3].

The growth in the number of children orphaned by HIV/AIDS continued to accelerate until 2000, when the number increased by 1.3 million. This population continued to grow, albeit more slowly, reaching 19.5 million in 2012 before declining to (a still staggering) 13.8 million in 2024 [3].

These celebrated achievements to fight the HIV threat for children and pregnant women occurred in the context of successful responses for HIV more generally, with overall reductions in new HIV acquisitions and deaths, improved life expectancy and stabilized economies. These achievements gave realistic hope that the threat of HIV to children could be eliminated—until the devastating actions by the U.S. government in January of 2025. The expected adverse impacts of those actions will be discussed elsewhere. Here, we will take stock of what have been the keys to success, define barriers to progress and needed changes, and, in the face of the current politically driven funding crisis, propose principles for a new road map to achieve our goal to eliminate HIV in children.

### 2.2 Key Factors Contributing to the Successful Paediatric HIV Response

Progress in protecting children from HIV has been the result of a remarkable combination of robust community and stakeholder engagement, science and innovation, data‐driven targets and monitoring, political commitment, multi‐level coordination and massive resource investment.

Organizations like EGPAF broadened their domestic campaign to a global mission to end AIDS in children through worldwide advocacy and service implementation [4]. Churches and faith‐based organizations were among the earliest and most vocal advocates for caring for children living with, orphaned by and otherwise affected by HIV in low‐ and middle‐income countries (LMICs), and they have remained a key and relentless voice for the global paediatric HIV response [5]. UNAIDS promoted the voices of women in driving progress for pregnant women, children and families. WHO, through its normative guidelines process and global accelerator for paediatric formulations (GAP‐f), highlighted and facilitated inclusion of pregnant women and children in HIV guidelines and optimal pediatric drug and formulation development [6, 7, 8, 9]. PEPFAR, the Global Fund and other donors similarly insisted on prioritizing pregnant women and children in programme planning and goal‐setting.

HIV research sponsored by the U.S. NIH and others enabled the HIV response to shift rapidly from palliative care and basic prevention to increasingly safe, efficacious, and pragmatic diagnostic, prevention, treatment and care interventions. Effective advocacy ensured that vertical transmission prevention and paediatric interventions were research priorities and the HIV field successfully promoted earlier, routine inclusion of pregnant women and children in clinical trials. The expertise and leadership for pregnancy and paediatric HIV research surged in the LMIC settings most affected by HIV.

Strong reliance on data to set ambitious targets and track progress has been a hallmark of the successful HIV response [10]. With key technical assistance from UNAIDS and donor programmes, national governments developed targets to reach vertical transmission and paediatric HIV goals and implemented robust epidemiological and programme data collection and monitoring. The use of data to inform progress and guide planning set a new standard for health programming.

The multi‐level, multi‐stakeholder, complex nature of protecting children from HIV has depended on robust coordination, political commitment and massive resource investment to succeed. In particular, the U.S. government designed the PEPFAR programme so that the State Department led diplomatic goal‐setting and joint political commitments with national governments and multilateral partners, directed implementation by health and development technical agencies (like CDC and USAID), and leveraged NIH and FDA processes to drive key innovations and endorse quality‐assured, safe and effective HIV medications for use in global programming [11]. The U.S. government invested billions of dollars per year in the HIV response for over 20 years, a major factor in the profoundly successful paediatric HIV achievements.

### 2.3 Paediatric HIV Elimination Progress Stagnated and Changes Were Overdue—Even Before the Political Funding Crisis of 2025

While the 2025 disruption of PEPFAR programme finances and operations has been destructive, the goal should not be merely to restore things to how they were. Many of the strategies and policies that were so effective in confronting the destabilizing HIV pandemic and accelerating life‐saving interventions globally are not what we need to close remaining gaps and solidify a sustainable, feasible end game.

The gaps are substantial. ART coverage for pregnant women has stalled and remains especially low (56%–65%) in lower HIV‐prevalence LMICs outside eastern and southern Africa [3]. A vertically oriented focus of HIV services for pregnant women in the highest‐burden areas was effective in the acceleration phase but has failed to reach the high numbers of women with HIV living outside those areas. PEPFAR continued to focus on more intensive maternal viral load testing and enhanced infant prophylaxis even though less than 10% of infant acquisitions occur when women remain on ART; more than 90% of infant acquisitions are the result of not reaching women with HIV prevention services (pre‐exposure prophylaxis [PrEP]), not reaching women with basic HIV testing and treatment, and failing to help women stay in care [3].

The same vertical, high‐burden area‐focused approach has contributed to stalled progress in paediatric HIV diagnosis and treatment. Many children reached with HIV services have died of infections and malnutrition in contexts where under‐5 mortality remains high and lacks adequate investment [12]. Sophisticated and innovative electronic medical record and data systems, client tracking systems and robust resources for HIV‐vulnerable children contributed to superior HIV programme and child outcomes—but in an unaffordable, unsustainable manner limited to HIV programming and benefitting only children and families affected by HIV.

The disproportionate and siloed resource investment for the HIV response is present at every level. UNAIDS was created as the only UN agency focused on a single disease. National governments created HIV‐specific “AIDS Councils” and similar structures that were highly funded and influenced by donors, often operating outside other programmes in the health ministry. PEPFAR and other donors tied their funding specifically to HIV work and expected national governments to implement plans frequently not well aligned with their health systems. Originator and generic drugmakers relied on USG funding to help develop and to buy HIV medications. While these HIV‐only investments helped launch and accelerate the successful HIV response, now is the time for transformation to an integrated, sustainable approach, less dependent on donor funding and designed to end HIV in children.

### 2.4 Getting Back on the Path to Eliminating HIV in Children

The following framework is proposed for achieving our commitment to children:

(1) *A fully integrated response can overcome stagnated paediatric HIV progress and improve all health outcomes for children and families*.

What remains of USAID global health programming is now part of the U.S. State Bureau of Global Health and Diplomacy (GHSD) that coordinates PEPFAR, presenting an opportunity for the U.S. Congress to provide authorization language for GHSD to coordinate all global health assistance and measure success by comprehensive maternal and child—not just HIV—outcomes. Political will seems supportive of a more integrated global health assistance approach [13]. Counterproductive USG interagency competition can be replaced with single‐agency leadership and accountability that draws strategically on other agency technical capacities (like the U.S. President's Malaria Initiative [PMI] model [14]). GAP‐f [9] is an excellent example of applying HIV lessons to create a model for accelerating optimal medication development across disease areas for children. UNAIDS could consider (1) transferring its robust HIV modelling and monitoring section to WHO to benefit all priority health areas and (2) broadening its essential community advocacy platform to promote progress for the overall health and welfare of children, pregnant women and others who remain vulnerable, stigmatized or forgotten. WHO and national programmes can reinforce the Making Pregnancy Safer and Integrated Management of Childhood Illness or similar strategies for implementation of evidence‐based, feasible, comprehensive maternal‐child health (MCH) interventions delivered at scale through national health systems and that sustainably incorporate HIV prevention and treatment services—including in countries that have not received donor HIV assistance. The shifts at these levels could ease distortions at the national health system and service delivery levels, facilitating more holistic MCH programming that can increase HIV (and other essential health) service access to more children and pregnant women.

(2) *National governments reassert leadership and accountability for the health of their people*.

Strong national government leadership has been essential for the successful HIV response and remarkable achievements. However, donor expectations that their priorities be accepted in return for funding hindered leadership and accountability of national governments, disincentivized growth in domestic funding for HIV, and often supported HIV systems and approaches that were not best aligned with the country health system context. The abrupt reduction in USG funding and influence reinforces the prime role of national governments in leading the effective integration of sustainable, high‐quality HIV services for children and families into strengthened national systems and investments for MCH at all service levels and across urban to rural settings [15]. It also reinforced the importance of national governments executing on and even increasing their funding commitments for HIV and public health. Global donors and other partner organizations remain important collaborators with national governments in the lead; cumbersome, complex, HIV‐focused data and service delivery systems may no longer be appropriate.

(3) *Donor funding and resources are prioritized for research and innovation*.

Current reductions in donor resources for the global paediatric HIV agenda challenge us to consider what donor investments will be most impactful and least likely funded by other sources. In place of historic predominant investments in health workforce, specialized digital tools and child/family social support, donor resources may be best prioritized for critical research that will drive further innovation in diagnosing, preventing and treating HIV. As LMICs struggle to assume funding for most HIV care (some low‐income countries will still need substantial programme funding assistance from donors for a successful transition), they will not likely have the resources to fund innovation by themselves. USG funding of research collaborations between American and LMIC academic organizations can produce innovations to end the threat of HIV in children globally while satisfying USG political objectives for building American capacity for MCH research and applying global innovations at home.

(4) *Public sector will rely more on strong partnerships with the pharmaceutical and private sector*

Most of the global HIV work has been financed and implemented with public sector resources (donor and domestic) through public sector health systems and facilities. The drugs, laboratory products and instruments, and digital tools that HIV programmes have procured with substantial donor subsidies have been produced by—and produced handsome profits for—private sector pharmaceutical (originator and generic), biomedical device and digital health companies. These businesses will no longer be able to count on donor funding across the product development cascade from research to regulatory approval and programme uptake. But private sector companies will still have an important and profitable role to play. They will likely streamline and focus product development areas in the face of reduced external partner funding and strengthen direct relationships and agreements with health leaders in target countries to advance development of products that health systems in those countries will want and use. Private sector delivery of health services could also increase access and reduce public sector burden [16]. Strong agreements that simultaneously satisfy company profit goals and serve population healthcare needs will be more sustainable than the current donor dependence. Continued technical expert and civil society organization (CSO) engagement can ensure products and services for children and pregnant women are properly considered—such as the real potential for broad use of lenacapavir to slash new HIV acquisitions in women (especially adolescent/young women) and infants [17].

*(5) Community voices and needs are amplified*.

The role of community advocacy in the success of the HIV response, domestically and globally, cannot be overstated. Decades ago, those community voices powerfully and effectively focused governments, societies and billions of dollars on responding to the HIV pandemic emergency. Fresh approaches to advocacy and communication campaigns that can motivate governments and communities towards action and hold each other accountable will be essential to ensure progress for the many children and families who cannot make themselves heard. A person‐ (rather than disease‐) centred UNAIDS [18] and faith‐based organizations [19] are well placed to amplify the voices of children and families and advocate not just for a narrow HIV agenda but for their broader health needs. The path to ending the threat of HIV in children will be through a robust general MCH agenda.

(6) *Creative thinking and redoubled determination are essential*.

The abrupt withdrawal of USG support could demoralize and discourage us from our work—but the world has faced difficult and desperate moments in the HIV epidemic before. That did not stop churches from caring for children stricken or affected by HIV/AIDS. That did not stop researchers from promoting trials of groundbreaking HIV interventions in pregnant women and children. That did not stop global coalitions of implementers, multilateral organizations and donors from advancing the HIV response for children and families. That did not stop communities affected by HIV from insisting that the world pay attention, devote resources and do better for ending the HIV threat to children. Once again, in this dark time, the diverse and talented global HIV stakeholder coalition will commit to protect and build on tremendous progress, find the opportunities for transformation and innovation in paediatric HIV care, and boldly chart a lasting, evidence‐based, person‐centred, community‐informed, national health system‐aligned path to ending HIV as a threat to children.

## 3 Conclusions

The global paediatric HIV community can turn the adversity of financial and political upheaval into an opportunity to assess the key factors that have enabled the tremendous progress towards ending the threat of HIV in children, define the critical barriers to overcoming remaining gaps and create a financially sustainable framework no longer so dependent on donor support for getting back on the path to eliminating HIV in children. Let us channel our righteous anger into bold, transformative actions that will leave no child behind—children are counting on us!

## Author Contributions

The author declares full and sole responsibility for the preparation of the manuscript.

## Funding

The author has nothing to report.

## Conflicts of Interest

The author has no conflicts of interest or competing interests to declare.

## Data Availability

Data sharing is not applicable to this commentary, for which no datasets were generated or analysed.
